# Insulin analogues in children with Type 1 diabetes: a 52-week randomized clinical trial

**DOI:** 10.1111/dme.12041

**Published:** 2013-01-21

**Authors:** N Thalange, A Bereket, J Larsen, L C Hiort, V Peterkova

**Affiliations:** 1 Jenny Lind Children's Department, Norfolk and Norwich University HospitalNorwich, UK; 2 Division of Paediatric Endocrinology and Diabetes, Marmara University School of MedicineIstanbul, Turkey; 3 Insulin Medical and Science, Novo Nordisk A/SBagsvaerd, Denmark; 4 Biostatistics, Novo Nordisk A/SBagsvaerd, Denmark; 5 Endocrinological Research Centre, Institute of Paediatric EndocrinologyMoscow, Russia

## Abstract

**Aims** This 52-week, randomized, multinational, open-label, parallel-group, non-inferiority trial investigated the efficacy and safety of basal–bolus treatment with insulin detemir vs. NPH (neutral protamine Hagedorn) insulin, in combination with insulin aspart, in subjects aged 2–16 years with Type 1 diabetes mellitus.

**Methods** Of the 347 randomized and exposed subjects, 177 received insulin detemir and 170 NPH insulin, both administered once or twice daily in combination with mealtime insulin aspart. Glycaemic measurements and weight were followed over 52 weeks.

**Results** After 52 weeks, insulin detemir was shown to be non-inferior to NPH insulin with regard to HbA_1c_ [mean difference insulin detemir–NPH: 1.30 mmol/mol, 95% CI –1.32 to 3.92 (0.12%, 95% CI –0.12 to 0.36) in the full analysis set and 1.41 mmol/mol, 95% CI –1.26 to 4.08 (0.13%, 95% CI –0.12 to 0.37) in the per protocol analysis set]. Hypoglycaemic events per subject-year of exposure of 24-h and nocturnal hypoglycaemia were significantly lower with insulin detemir than with NPH insulin (rate ratio 0.76, 95% CI 0.60–0.97, *P* = 0.028 and 0.62, 95% CI 0.47–0.84, *P* = 0.002, respectively). Weight standard deviation (sd) scores (body weight standardized by age and gender) decreased with insulin detemir, but increased slightly with NPH insulin (change: –0.12 vs. 0.04, *P* < 0.001). At end of the trial, median insulin doses were similar in both treatment groups.

**Conclusions** Insulin detemir was non-inferior to NPH insulin after 52 weeks' treatment of children and adolescents aged 2–16 years, and was associated with a significantly lower risk of hypoglycaemia, together with significantly lower weight sd score when compared with NPH insulin.

## Introduction

Evidence from the Diabetes Control and Complications Trial (DCCT) and other landmark studies 1–4, have shown that tight glycaemic control in adults and adolescents with Type 1 diabetes mellitus is of crucial importance in reducing the premature onset of micro- and macrovascular complications 3–6. However, in these studies, intensive insulin therapy was associated with an increased risk of hypoglycaemia and increased body weight. These studies did not include pre-adolescent children, but other studies have confirmed that poor glycaemic control in the young is not harmless 7. For example, periods of hyperglycaemia and episodes of severe hypoglycaemia are associated with impaired cognitive and intellectual development in children with Type 1 diabetes 8,9.

The challenge of achieving good glycaemic control is more difficult in children and adolescents compared with adults because of the characteristics of the paediatric population. Growth, variable exercise and eating patterns, dependence on parents and other caregivers for injections and blood tests, difficulties ensuring optimal diabetes care in schools and the physiological and psychological burdens of adolescence all constitute challenges to achieving optimal glycaemic control 10. Consequently, an ideal insulin regimen would be flexible and predictable, whilst protecting against hypoglycaemia 11 and inappropriate weight gain.

Insulin detemir is a long-acting, soluble acylated analogue of human insulin [Lys^B29^ (N^ε^tetradecanoyl) des (B30) human insulin] with a protracted action profile attributable to increased self-association at the injection site and buffering of insulin concentration via albumin binding in both the subcutaneous tissue and the blood 12. In contrast to neutral NPH human isophane insulin 13,14, insulin detemir does not require re-suspension before injection.

Clinical trials in adults with Type 1 diabetes have shown that insulin detemir is associated with comparable HbA_1c_, less variability in fasting plasma glucose, less nocturnal hypoglycaemia and less weight gain compared with intermediate-acting NPH 15–18. In spite of the importance of optimizing diabetes care in children, few comparative randomized clinical trials have been conducted in this age group.

The aim of the current study was to establish the efficacy and safety of insulin detemir in children aged 2–16 years with Type 1 diabetes over 52 weeks of treatment. Results for the youngest age strata (2–5 years) have already been published, and it was found that insulin detemir was associated with similar glycaemic control, but a greater reduction in fasting plasma glucose and a lower rate of hypoglycaemia when compared with NPH insulin in these patients after 52 weeks 19.

## Subjects and methods

### Subjects

Children with Type 1 diabetes for at least 12 months (*n* = 347), aged 2–16 years (~60% prepubertal and ~40% pubertal), a total daily insulin dose ≤ 2.0 U/kg, with HbA_1c_ ≤ 97 mmol/mol (11.0%), insulin detemir-naïve, non-obese (maximum BMI ≤ 27 kg/m^2^, depending on age) were recruited from diabetes clinics at 35 sites in 11 European countries (Bulgaria, the Czech Republic, Denmark, Finland, France, Hungary, Macedonia, Poland, Russia, Turkey and the UK). Subjects with clinically significant concomitant diseases (as judged by the investigator) were excluded. The study was approved by local ethics committees and health authorities, and carried out in accordance with Good Clinical Practice 20 and the Declaration of Helsinki 21. Written informed consent was obtained from all children (where appropriate) and their parents or legal representative before any study-related activities.

### Study design

In this 52-week, multinational, open-label, randomized (1:1, insulin detemir:NPH), two-armed parallel-group trial, eligible children were stratified according to age; 2–5 years (*n* = 82, 23.6%) and 6–16 years (*n* = 265, 76.4%). In addition, in a post-hoc statistical analysis, the 6- to 16-year-old cohort was separated into 6- to 12-year-old and 13- to 16-year-old subgroups. Insulin detemir and NPH were administered once or twice daily (according to pretrial regimen), and both treatment groups received insulin aspart as bolus insulin with main meals and larger snacks. The trial consisted of a 2-week screening period, followed by a 52-week titration and treatment period, including a total of 10 scheduled visits and eight telephone contacts during the treatment period. Eligibility for the study was confirmed at an initial screening visit. Eligible subjects were allocated to treatment with insulin detemir or NPH (1:1). Randomization was carried out using a centralized telephone and web-based randomization system, the Interactive Voice Response System (IVRS)/Interactive Web Response System (IWRS), performed within 2 weeks of the screening visit. Insulin detemir and NPH are visually distinguishable and, as the primary endpoint HbA_1c_ is not easily biased, an open-label study design was chosen. Stringent glycaemic control in children is often avoided in real-life clinical practice because of potential hypoglycaemia and its negative effects on young patients. So that the diabetes treatment given during this trial could closely resemble a real-life environment, this study was not a treat-to-target design.

### Treatment

Children were treated with insulin detemir (Levemir®; Novo Nordisk A/S, Bagsvaerd, Denmark; 100 U/ml) or NPH (NPH, human isophane insulin®; Novo Nordisk A/S; 100 IU/ml) once or twice daily, according to pretrial insulin regimen and dose. Both groups received insulin aspart (NovoRapid®/NovoLog®; Novo Nordisk A/S; 100 U/ml) 2–4 times daily, with main meals.

During the treatment period, the basal insulin dose was adjusted according to plasma glucose measurements with a preprandial plasma glucose goal of 4.0–7.0 mmol/l (72–126 mg/dl). Participants were asked to measure their plasma glucose before breakfast and dinner on the last 3 days prior to each contact and to adjust basal insulin doses according to a simple titration algorithm (Table 1).

**Table 1:** Algorithm for titration of basal insulin dose

Current dose		< 5 U	5–15 U	> 15 U
Pre-breakfast or pre-dinner plasma glucose		Adjustment (U)		
< 4.0 mmol/l	< 72 mg/dl	Reduce according to local practice	Reduce according to local practice	Reduce according to local practice
4.0–7.0 mmol/l	72–126 mg/dl	0	0	0
7.1–10.0 mmol/l	127–180 mg/dl	+0.5	+1	+2
10.1–15.0 mmol/l	181–270 mg/dl	+1	+2	+4
> 15.0 mmol/l	> 270 mg/dl	+1.5	+3	+5

Bolus insulin was to be taken 0–15 min prior to or immediately after the meal, aiming at a postprandial plasma glucose of 5.0–11.0 mmol/l (90–198 mg/dl) measured 1–4 h after a meal. Bolus insulin doses were adjusted according to local practice.

### Efficacy measures

The primary endpoint of this trial was HbA_1c_ measured after 52 weeks. Blood samples for HbA_1c_ and fasting plasma glucose were drawn at randomization and approximately every 3 months subsequently. Nine-point self-monitored plasma glucose profiles, including nocturnal plasma glucose at 03.00 h, were assessed by the children on a normal weekday 4–7 days prior to randomization, and after 26 and 52 weeks of treatment.

Hypoglycaemic episodes were included as a secondary endpoint of this trial and were classified according to International Society for Pediatric and Adolescent Diabetes (ISPAD) guidelines 22,23. Mild hypoglycaemia was defined as episodes where the subjects were able to treat themselves. Moderate hypoglycaemia was categorized as episodes where subjects required assistance, but responded to oral treatment. Severe hypoglycaemia was defined as episodes where the subjects were semi-conscious, unconscious or in a coma, with or without convulsions. Episodes without signs/symptoms but with plasma glucose value < 3.6 mmol/l (< 65 mg/dl) were defined as biochemical hypoglycaemia.

### Body weight

Body weight was determined as a secondary endpoint and was measured at each visit and subsequently analysed by standard deviation (sd) scores in order to compare children of different age and gender, using British growth standards 24. A positive sd score indicates a higher weight level compared with the population mean for a given age.

### Safety measures

Standard safety variables including adverse events, laboratory analyses (haematology and biochemistry), physical examination findings and vital signs were evaluated as secondary outcomes of the trial. Serious adverse events were defined as, amongst others, a life-threatening experience, inpatient hospitalization or prolongation of existing hospitalization, a persistent or significant disability/incapacity or death.

Fundoscopy/fundus photography, physical examination and vital signs were evaluated at randomization and after 52 weeks of treatment. Height measured in centimetres was recorded pretreatment and after 26 and 52 weeks of treatment. Pubertal status was graded as prepubertal (Tanner grade 1) or pubertal (Tanner grade 2 or more) 1986 pretreatment and after 52 weeks.

### Analytical methods

HbA_1c_ measurements and other laboratory analyses were performed centrally by the Laboratorium für Klinische Forschung (Germany). HbA_1c_ was measured by ion-exchange high-performance liquid chromatography (HPLC) (Bio-Rad Diamat™; Bio-Rad Laboratories, Hercules, CA, USA) and fasting plasma glucose values were assessed using a hexokinase method (Gluco-quant®; Roche Diagnostics GmbH, Mannheim, Germany). Self-monitored plasma glucose was measured using a glucose meter (Medisense Precision Xtra™ or Optimum Plus™; Abbott Diabetes Care, Delkenheim, Germany). Use of test strips calibrated to plasma glucose values ensured that capillary blood concentrations were displayed as plasma glucose values. All self-monitored plasma glucose values < 3.6 mmol/l (< 65 mg/dl), as well as signs and symptoms of hypoglycaemia, were recorded in the patients' diaries and included in the analyses of hypoglycaemia. All blood samples were obtained in the morning before administration of insulin.

### Statistical analysis

The trial was designed to confirm efficacy of insulin detemir in children in terms of glycaemic control. This was carried out by showing that insulin detemir was non-inferior to NPH both in combination with insulin aspart with respect to HbA_1c_ after 52 weeks of treatment using a non-inferiority criterion of 0.4% 26. Throughout the analyses, a significance level of 5% was used.

The power calculation was analysed on this basis: using a two-sided *t*-test with a one-sided significance level of 2.5%, assuming sd of 1.1, a non-inferiority criterion of 0.4%, a power of 85% and an expected dropout rate of 20%, a total of 344 children were to be randomized.

The primary endpoint HbA_1c_ at the end of the trial was tested for non-inferiority using a normal linear regression model with treatment, pubertal status at baseline, country and age stratification at randomization (only mentioned as age in the remaining part of the section) as factors, and baseline HbA_1c_ as covariate. The primary efficacy analysis was based on both the full analysis set and per protocol population. The full analysis set was defined as all randomized subjects exposed to at least one dose of trial product with a post-baseline observation, classified according to randomized treatment. The per protocol population was defined as all exposed subjects who took part in the trial without significantly violating the inclusion/exclusion criteria or other aspects of the protocol considered to potentially affect the efficacy results, classified according to actual treatment.

Fasting plasma glucose at the end of the trial was analysed using a similar model, replacing baseline HbA_1c_ with baseline fasting plasma glucose. Last observation carried forward was applied to all HbA_1c_ and fasting plasma glucose post-randomization values. All secondary efficacy analyses were based on the full analysis set.

Within-subject variation of self-monitored plasma glucose was estimated using a variance-component model with country, treatment and age as fixed factors and subject as random effect. The test was carried out as a likelihood ratio test comparing the model with a separate residual variance component for each treatment group to a model with a common residual variance component.

Nine-point self-monitored plasma glucose profiles were analysed using a repeated-measurements model with treatment, country, pubertal status at baseline, age, time point and the treatment by time-point interaction as fixed factors. The covariance structure was modelled as unstructured for each subject and independence between subjects was assumed.

The number of hypoglycaemic episodes per subject was analysed using a negative binomial regression model with treatment and age as factors and the logarithm of subject exposure as offset, and from this model the rate ratio between the two treatment groups was estimated. This was performed for nocturnal, diurnal and 24-h hypoglycaemic episodes divided in the categories All, Severe, Moderate, Mild and Biochemical. Hypoglycaemic episodes were categorized as nocturnal if they occurred between 22.00 and 07.00 h.

The weight sd score at the end of the trial was analysed with a normal linear regression model with treatment, age and country as factors and baseline sd score as a covariate. Last observation carried forward was applied to all post-randomization weight values.

## Results

### Demographics

**Figure 1:**
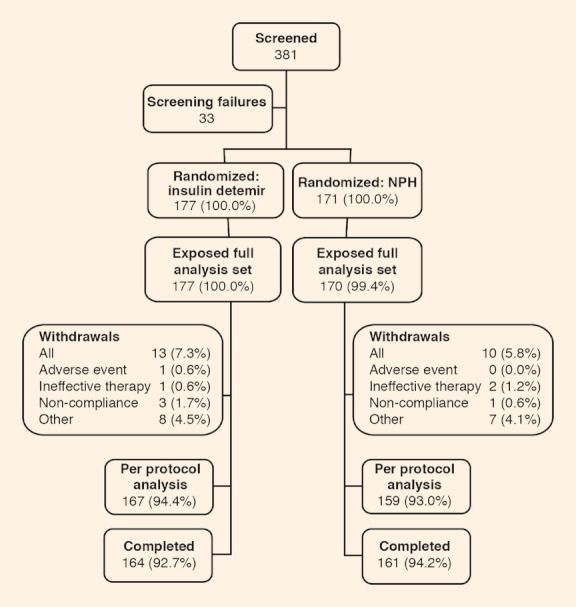
Subject disposition. Per protocol analysis set was defined as children not significantly violating the inclusion/exclusion criteria or other aspects of the protocol considered to potentially affect the efficacy results with an exposure of at least 6 months. ‘Other' included unspecified unwillingness to continue (14 subjects) and high level of HbA_1c_ (one subject). NPH, neutral protamine Hagedorn.

A total of 381 children were screened, of whom 33 failed to meet all the selection criteria, the majority because of HbA_1c_ > 97 mmol/mol (> 11%) (Fig. 1). Three hundred and forty-eight children entered the trial, of whom 347 were exposed to trial products. One subject allocated to NPH withdrew consent prior to treatment. The treatment groups were similar with respect to withdrawal and baseline characteristics (Table 2). The median daily dose of basal, bolus and premixed insulin per kg body weight was similar in the two groups.

**Table 2:** Baseline characteristics

Subjects exposed to treatment, *n* (%)	Insulin detemir 177 (100.0%)	NPH 170 (99.4%)	All 347 (99.7%)
Gender			
Female	94 (53.1%)	73 (42.9%)	167 (48.1%)
Male	83 (46.9%)	97 (57.1%)	180 (51.9%)
Age (years)	10.0 (4.09)	9.8 (3.90)	9.9 (3.99)
Pubertal status			
Tanner grade 1	104 (58.8%)	104 (61.2%)	208 (59.9%)
Tanner grade 2	73 (41.2%)	66 (38.8%)	139 (40.1%)
Stratification by age			
2–5 years	42 (23.7%)	40 (23.5%)	82 (23.6%)
6–16 years	135 (76.3%)	130 (76.5%)	265 (76.4%)
Diabetes duration (years)	3.70 (2.66)	3.68 (2.51)	3.69 (2.58)
Body weight sd score	0.24 (0.92)	0.32 (0.99)	0.28 (0.96)
HbA_1c_ (mmol/mol)*	68.42 (12.1)	68.29 (12.0)	68.36 (12.1)
(%)*	8.41 (1.11)	8.40 (1.10)	8.40 (1.10)
Fasting plasma glucose (mmol/l)*	8.36 (4.38)	8.70 (4.59)	8.52 (4.48)
Pretrial insulin regimens			
1 basal + 3 bolus injections daily	40%	34%	
2 basal + 3 bolus injections daily	25%	31%	
Premix alone daily	8%	9%	
Premix + basal and/or bolus daily	14%	15%	
Pretrial daily insulin dose			
Basal insulin (IU/kg)	0.43 (0.09–1.22)	0.47 (0.03–2.82)	0.45 (0.03–2.82)
Mealtime insulin (U/kg)	0.46 (0.07–1.00)	0.44 (0.02–1.00)	0.45 (0.02–1.00)
Premix	0.80 (0.05–5.75)	0.62 (0.01–1.17)	0.70 (0.01–5.75)

Numbers are *n* (%), mean (standard deviation) or median (range).

* HbA_1c_, fasting plasma glucose recorded at or before randomization.

NPH, neutral protamine Hagedorn.

### Glycaemic control

**Figure 2:**
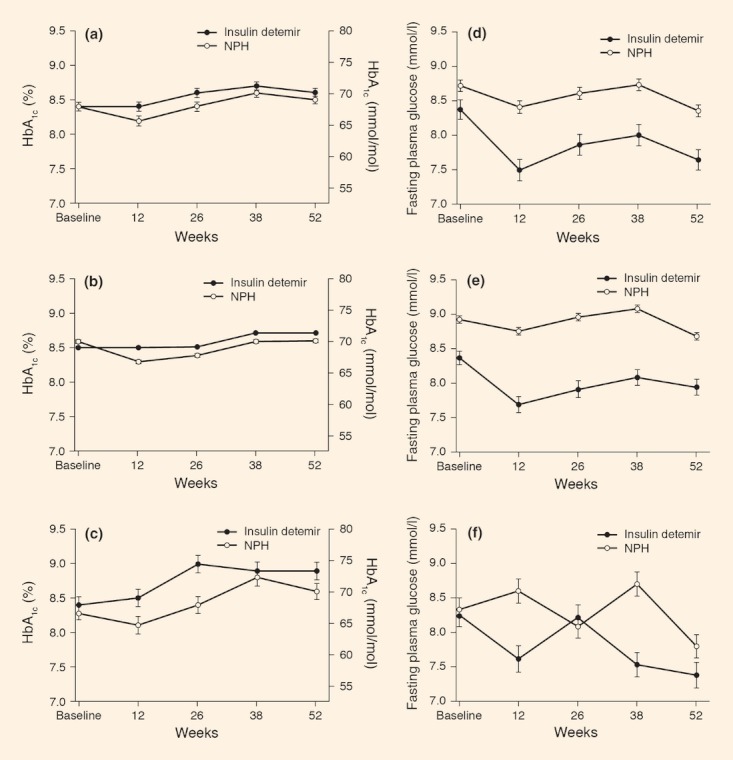
Observed mean glycated haemoglobin (HbA_1c_) (± sem) over time for full analysis set last observation carried forward for the overall cohort (a), the 6- to 12-year-old subgroup (b) and the 13- to 16-year-old cohort (c), and fasting plasma glucose (± sem) over time for full analysis set last observation carried forward for the overall cohort (d), the 6- to 12-year-old subgroup (e) and the 13- to 16-year-old cohort (f). NPH, neutral protamine Hagedorn.

After 52 weeks, non-inferiority of insulin detemir plus insulin aspart vs. NPH plus insulin aspart was established for HbA_1c_ in the overall population [full analysis set: insulin detemir 72.18 mmol/mol (8.75%), NPH 70.88 mmol/mol (8.64%), mean difference insulin detemir–NPH 1.30 mmol/mol, 95% CI –1.32 to 3.92 (0.12%, 95% CI –0.12 to 0.36%); and per protocol: insulin detemir 72.13 mmol/mol (8.75%), NPH 70.72 mmol/mol (8.62%), mean difference insulin detemir–NPH 1.41 mmol/mol, 95% CI –1.26 to 4.08 (0.13%, 95% CI –0.12 to 0.37%)]. HbA_1c_ levels slightly and gradually increased over 52 weeks—both in patients given insulin detemir and those receiving NPH (Fig. 2a). The highest HbA_1c_ was measured at week 38 for both treatments {mean (sd) NPH: 70.2 (13.8) mmol/mol [8.6 (1.3)%]; insulin detemir: 71.1 (16.0) mmol/mol [8.7 (1.5)%]}.

Given the physiological differences in children across the 6- to 16-year age range, we also analysed the data as two age groups: 6–12 years (*n* = 167) and 13–16 years (*n* = 98). There was a slight increase in HbA_1c_ with both regimens for the 6- to 12-year-old and 13- to 16-year-old subgroups over time (Fig. 2b and c). Mean HbA_1c_ remained stable with both treatments in the 2- to 5-year-old cohort 19.

Both regimens appeared to cause a fairly rapid drop in fasting plasma glucose at week 12, followed by a slight increase measured at weeks 24 and 36 and a final decrease between weeks 36 and 52 in the overall population (Fig. 2d). Although the mean change in fasting plasma glucose tended to be more pronounced with insulin detemir (–0.60 mmol/l) than with NPH (0.02 mmol/l), no significant difference was observed after 52 weeks. Mean difference (insulin detemir–NPH) was –0.63 mmol/l, 95% CI –1.56 to 0.31 (Fig. 2d). Mean fasting plasma glucose levels had a net decrease from baseline to end of trial in both the 6- to 12-year-old and the 13- to 16-year-old cohorts for both treatments (Fig. 2e and f). In the 6- to 12-year-old cohort, fasting plasma glucose was consistently lower with insulin detemir than NPH throughout the trial (Fig. 2e), whereas fasting plasma glucose was comparable between treatments in the 13- to 16-year-old cohort over time (Fig. 2f). In the cohort of 2–5-year-old children fasting plasma glucose was similar at baseline and decreased with both therapies during the study 19.

Within-subject variation in fasting plasma glucose measurements assessed by self-monitored plasma glucose at 52 weeks was lower with insulin detemir than with NPH in the total cohort (sd 3.01 vs. 3.68 mmol/l, *P* < 0.001).

The analysis of nocturnal plasma glucose (03.00 h) after 52 weeks showed no significant difference between treatments in the overall population. The total median daily insulin dose per kg body weight was similar in the two treatment groups after 52 weeks for all subgroups and the total cohort (Table 3) 19. The ratios (insulin detemir:NPH) of the median daily insulin doses at the end of the trial were 1.04 (0.57:0.55 U/kg) for basal insulin and 1.09 (0.47:0.43 U/kg) for bolus insulin.

**Table 3:** Daily insulin dose

Insulin dose	6–12 years old		13–16 years old		Total cohort	
Insulin detemir	NPH	Insulin detemir	NPH	Insulin detemir	NPH
Basal insulin						
Baseline	0.48	0.44	0.41	0.44	0.42	0.42
End of trial	0.64	0.63	0.53	0.54	0.57	0.55
Bolus insulin						
Baseline	0.42	0.46	0.52	0.47	0.46	0.46
End of trial	0.44	0.40	0.48	0.47	0.47	0.43

Data are median, in U/kg.

NPH, neutral protamine Hagedorn.

### Hypoglycaemic episodes

**Figure 3:**
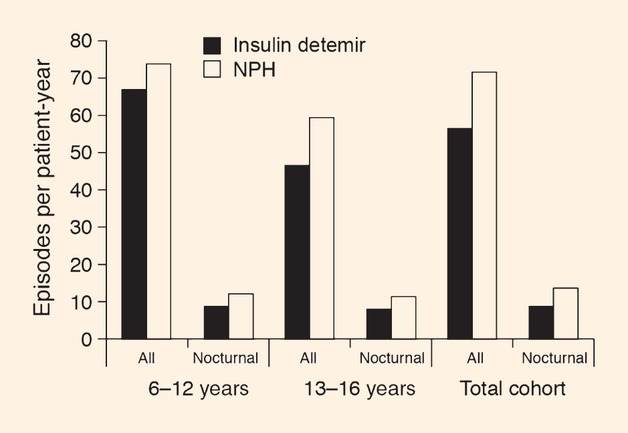
The mean rate of hypoglycaemic episodes (per patient-year) in 6- to 12-year-old subgroup, 13- to 16-year-old subgroup and the total cohort. Nocturnal hypoglycaemia: 22.00–07.00 h, inclusive. NPH, neutral protamine Hagedorn.

In the total cohort, children treated with insulin detemir had a lower rate of episodes than those treated with NPH (Table 4). No severe nocturnal hypoglycaemic episodes were reported in the insulin detemir group, while five episodes took place in the NPH group (Table 4). The rate of all 24-h episodes, mild 24-h episodes, any nocturnal episodes and mild nocturnal episodes was lower with insulin detemir than with NPH insulin in the overall population (Table 4, Fig. 3). This was also observed in the separate 6- to 12-year-old and 13- to 16-year-old subgroups (Fig. 3), and frequency of hypoglycaemia was numerically lower in the 13- to 16-year-olds compared with the 6- to 12-year-old subgroup (Fig. 3). In the 2- to 5-year-old cohort, a lower rate of hypoglycaemia was observed with insulin detemir compared with NPH 19.

**Table 4:** Summary of all hypoglycaemic episodes

	Insulin detemir				NPH							
*n*	(%)	Episodes	Rate*	*n*	(%)	Episodes	Rate*	Rate ratio†	95% CI	*P*-value
All 24-h episodes	168	95	9448	56.3	166	98	11 576	71.0	0.76	0.60–0.97	0.028
Severe 24 h	3	2	3	0.0	12	7	15	0.1	—	—	—
Moderate 24 h	30	17	370	2.2	28	16	947	5.8	0.38	0.14–1.03	0.057
Mild 24 h	148	84	5956	35.5	151	89	7189	44.1	0.71	0.52–0.96	0.027
Biochemical 24 h	135	76	3119	18.6	128	75	3425	21.0	0.90	0.63–1.29	0.565
All nocturnal	131	74	1379	8.2	141	83	2141	13.1	0.62	0.47–0.84	0.002
Severe nocturnal	0	0	0	0.0	5	3	6	0.0	—	—	—
Moderate nocturnal	15	8	59	0.4	14	8	112	0.7	0.44	0.14–1.42	0.169
Mild nocturnal	100	56	712	4.2	111	65	1139	7.0	0.54	0.38–0.78	0.001
Biochemical nocturnal	83	47	608	3.6	85	50	884	5.4	0.74	0.47–1.17	0.194
All diurnal	167	94	8069	48.1	166	98	9435	57.9	0.79	0.62–1.01	0.061
Severe diurnal	3	2	3	0.0	8	5	9	0.1	—	—	—
Moderate diurnal	27	15	311	1.9	27	16	835	5.1	0.37	0.13–1.03	0.058
Mild diurnal	146	82	5244	31.2	150	88	6050	37.1	0.74	0.55–1.01	0.060
Biochemical diurnal	128	72	2511	15.0	123	72	2541	15.6	0.95	0.66–1.37	0.783

* Number of episodes per subject-year of exposure.

† Adjusted rate from negative binomial regression model including log-transformed exposure time as an offset variable, stratification and treatment.

NPH, neutral protamine Hagedorn.

### Mean sd score of body weight

**Figure 4:**
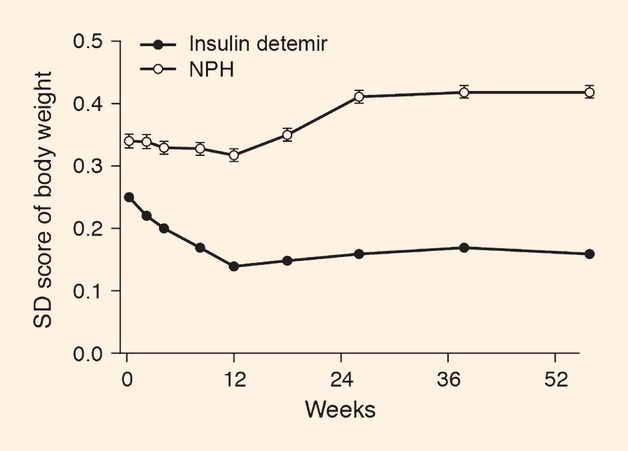
Observed mean weight sd score over time for safety analysis set last observation carried forward. NPH, neutral protamine Hagedorn.

The difference between the two treatment groups in estimated weight sd score at the end of the trial was significant for the overall population (insulin detemir 0.18, NPH 0.33, mean difference –0.15, 95% CI –0.23 to –0.07, *P* < 0.001) (Fig. 4).

### Adverse events

For the total cohort, the number of events/1000 exposure-years of all adverse events and non-serious adverse events were similar in the two treatment groups, while the rate of serious adverse events was lower with insulin detemir (95) than with NPH (147) (see also Supporting Information, Table S1).

## Discussion

To our knowledge, this study is the largest and longest randomized clinical trial ever performed in children and adolescents with Type 1 diabetes.

The efficacy results from the trial showed that glycaemic control with insulin detemir plus insulin aspart was non-inferior to treatment with NPH plus insulin aspart, as measured by the primary endpoint HbA_1c_ after 52 weeks of treatment. The slight increase in HbA_1c_ seen with insulin detemir and NPH reflects the difficulties in treating children for whom many factors, including social status, diabetes care in school or day care, highly variable lifestyle and (fear of) hypoglycaemia, influence glycaemic control 8. Additionally, physicians and families are often reluctant to titrate insulin in children as aggressively as in adults, because of the risk of severe hypoglycaemia and its consequences 27.

Another finding similar to previously reported results in both children and adults was that of a lower within-subject variation in self-monitored plasma glucose before breakfast with insulin detemir than with NPH 28–30. The low glucose-lowering variability of an insulin is desirable, as fluctuations in glucose levels have been shown to increase the risk of retinopathy and neuropathy in patients with Type 1 diabetes and may induce higher rates of hypoglycaemia 31.

Insulin detemir was associated with a significantly lower risk of 24-h and nocturnal hypoglycaemia. The lower rate of hypoglycaemia with insulin detemir may in part be attributable to its lower within-subject variability compared with NPH. These results support findings from a similar study also examining insulin detemir with insulin aspart with children with Type 1 diabetes 30. Severe hypoglycaemia is associated with impaired cognitive capabilities in children 8,9 and parents' fear of hypoglycaemia episodes has been shown to impact negatively on adherence to diabetes treatment and thus glycaemic control 32. Hence, attempting to minimize the occurrence of hypoglycaemia in children is essential, and it is therefore noteworthy that there were fewer severe hypoglycaemic events with insulin detemir than with NPH.

Taken together, the outcomes discussed above suggest a reduction in hypoglycaemia was achieved with insulin detemir vs. NPH without an increase in HbA_1c_ and, reassuringly, this was seen across all age groups studied 19.

Weight gain was also lower for insulin detemir when compared with NPH for all age strata. The decrease in weight sd score from baseline to end of trial in those children treated with insulin detemir indicated that their weight decreased towards that for the normal reference population. Accurate and detailed growth standards were not available for all 11 participating countries; accordingly, British standards 23 were used. These standards allow very accurate calculation of sd score as data are available for monthly time intervals and gender. This was not the case for other available population data. Although the derivation of the sd score for all countries was based on British growth curves, the difference between treatment arms was not influenced by this.

The weight-sparing property of insulin detemir is a phenomenon observed in several other studies in both Type 1 28 and Type 2 diabetes 33, but the reasons for this remain unclear. The importance of keeping weight gain associated with diabetes therapy low, especially in adolescents, is highlighted by the observation that young people with Type 1 diabetes often use insulin omission as a strategy to manage their weight 34,35, with a detrimental effect on glycaemic control, and complication risk.

In consequence of the findings of this trial, regulatory approval has been granted by the European Medicines Agency for use of insulin detemir within the European Union, for children aged 2–5 years.

In conclusion, this study confirmed efficacy of insulin detemir by demonstrating non-inferiority of insulin detemir compared with NPH with respect to HbA_1c_, with an improved safety profile including significantly fewer hypoglycaemic episodes and less undesirable weight gain in children and adolescents aged 2–16 years with Type 1 diabetes. An ideal insulin regimen—particularly for children—should be physiological, flexible and predictable, whilst protecting against hypoglycaemia and inappropriate weight gain. Although here the efficacy and tolerability of insulin detemir was compared with NPH, which is not always used as part of a basal–bolus regimen in the young in clinical practice, the most suitable comparator, insulin glargine, has not yet been approved for use in children under the age of 6 years, and therefore the choice of NPH as a comparator does not detract from the positive outcomes seen with insulin detemir. This study therefore confirms that insulin detemir, coupled with insulin aspart in a basal–bolus insulin regimen, has the key characteristics of an ideal insulin regimen, and that these benefits extend to the very young.

## Funding sources

This study was supported financially by Novo Nordisk A/S, Denmark.

## Competing interests

This study was supported financially by Novo Nordisk A/S, Denmark, who were also responsible for the design, conduct, analysis and reporting of the study, with input from the authors. All authors were involved in the preparation and approval of the manuscript in collaboration with Novo Nordisk. NT has received fees for speaking and consulting from Novo Nordisk A/S. LCH and JL are employed by, and hold stock in, Novo Nordisk A/S. AB and VP have no competing interests to declare.

## Supporting Information

Table S1 Summary of adverse events.

## References

[b1] Wang PH, Lau J, Chalmers TC (1993). Meta-analysis of effects of intensive blood-glucose control on late complications of type I diabetes. Lancet.

[b2] The Diabetes Control and Complications Trial Research Group (1996). The absence of a glycemic threshold for the development of long-term complications: the perspective of the Diabetes Control and Complications Trial. Diabetes.

[b3] Reichard P, Nilsson BY, Rosenqvist U (1993). The effect of long-term intensified insulin treatment on the development of microvascular complications of diabetes mellitus. N Engl J Med.

[b4] The Diabetes Control and Complications Trial Research Group (1993). The effect of intensive treatment of diabetes on the development and progression of long-term complications in insulin-dependent diabetes mellitus. N Engl J Med.

[b5] Nathan DM, Cleary PA, Backlund JY, Genuth SM, Lachin JM, Orchard TJ (2005). Intensive diabetes treatment and cardiovascular disease in patients with type 1 diabetes. N Engl J Med.

[b6] Dahl-Jorgensen K, Brinchmann-Hansen O, Hanssen KF, Sandvik L, Aagenaes O (1985). Rapid tightening of blood glucose control leads to transient deterioration of retinopathy in insulin dependent diabetes mellitus: the Oslo study. Br Med J (Clin Res Ed).

[b7] Amin R, Widmer B, Prevost AT, Schwarze P, Cooper J, Edge J (2008). Risk of microalbuminuria and progression to macroalbuminuria in a cohort with childhood onset type 1 diabetes: prospective observational study. Br Med J.

[b8] Perantie DC, Lim A, Wu J, Weaver P, Warren SL, Sadler M (2008). Effects of prior hypoglycemia and hyperglycemia on cognition in children with type 1 diabetes mellitus. Pedriatr Diabetes.

[b9] Gonder-Frederick LA, Zrebiec JF, Bauchowitz AU, Ritterband LM, Magee JC, Cox DJ (2009). Cognitive function is disrupted by both hypo- and hyperglycemia in school-aged children with type 1 diabetes: a field study. Diabetes Care.

[b10] Silverstein J, Klingensmith G, Copeland K, Plotnick L, Kaufman F, Laffel L (2005). Care of children and adolescents with type 1 diabetes. Diabetes Care.

[b11] Ludvigsson J, Bolli GB (2001). Intensive insulin treatment in diabetic children. Diabetes Nutr Metab.

[b12] Havelund S, Plum A, Ribel U, Jonassen I, Volund A, Markussen J (2004). The mechanism of protraction of insulin detemir, a long-acting, acylated analog of human insulin. Pharm Res.

[b13] Heise T, Nosek L, Rønn BB, Endahl L, Heinemann L, Kapitza C (2004). Lower within-subject variability of insulin detemir in comparison to NPH insulin and insulin glargine in people with type 1 diabetes. Diabetes.

[b14] Jehle PM, Micheler C, Jehle DR, Breitig D, Boehm BO (1999). Inadequate suspension of neutral protamine Hagendorn (NPH) insulin in pens. Lancet.

[b15] De Leeuw I, Vague P, Selam JL, Skeie S, Lang H, Draeger E (2005). Insulin detemir used in basal–bolus therapy in people with type 1 diabetes is associated with a lower risk of nocturnal hypoglycaemia and less weight gain over 12 months in comparison to NPH insulin. Diabetes Obes Metab.

[b16] Hermansen K, Fontaine P, Kukolja KK, Peterkova V, Leth G, Gall MA (2004). Insulin analogues (insulin detemir and insulin aspart) versus traditional human insulins (NPH insulin and regular human insulin) in basal–bolus therapy for patients with type 1 diabetes. Diabetologia.

[b17] Home P, Bartley P, Russell-Jones D, Hanaire-Broutin H, Heeg JE, Abrams P (2004). Insulin detemir offers improved glycemic control compared with NPH insulin in people with type 1 diabetes: a randomized clinical trial. Diabetes Care.

[b18] Russell-Jones D, Simpson R, Hylleberg B, Draeger E, Bolinder J (2004). Effects of QD insulin detemir or neutral protamine Hagedorn on blood glucose control in patients with type I diabetes mellitus using a basal–bolus regimen. Clin Ther.

[b19] Thalange N, Bereket A, Larsen J, Hiort LC, Peterkova V (2011). Treatment with insulin detemir or NPH insulin in children aged 2–5 years with type 1 diabetes mellitus. Pediatr Diabetes.

[b20] International Conference of Harmonisation (ICH) (1996). Topic E6: Harmonised Tripartite Guidelines for Good Clinical Practice, Step 5, Consolidated Guideline 1.5.96.

[b21] World Medical Association (WMA) Declaration of Helsinki: Ethical Principles for Medical Research Involving Human Patients, 52nd WMA General Assembly, Edinburgh, Scotland, October 2000 and last amended with Note of Clarification on Paragraph 29 by the WMA General Assembly 2002, Washington.

[b22] International Society for Pediatric and Adolescent Diabetes (ISPAD) Guidelines.

[b23] Clarke W, Jones T, Rewers A, Dunger D, Klingensmith GJ (2008). Assessment and management of hypoglycemia in children and adolescents with diabetes. Pediatr Diabetes.

[b24] Cole TJ, Freeman JV, Preece MA (1998). British 1990 growth reference centiles for weight, height, body mass index and head circumference fitted by maximum penalized likelihood. Stat Med.

[b25] Tanner JM (1986). Normal growth and techniques of growth assessment. Clin Endocrinol Metab.

[b26] Food and Drug Administration Code of Federal Regulations, Guidance for Industry, Diabetes Mellitus: Developing Drugs and Therapeutic Biologics for Treatment and Prevention. Draft Guidance.

[b27] Ryan C, Gurtunca N, Becker D (2005). Hypoglycemia: a complication of diabetes therapy in children. Pediatr Clin North Am.

[b28] Bartley PC, Bogoev M, Larsen J, Philotheou A (2008). Long-term efficacy and safety of insulin detemir compared to Neutral Protamine Hagedorn insulin in patients with Type 1 diabetes using a treat-to-target basal–bolus regimen with insulin aspart at meals: a 2-year, randomized, controlled trial. Diabet Med.

[b29] Vague P, Selam JL, Skeie S, De Leeuw I, Elte JW, Haahr H (2003). Insulin detemir is associated with more predictable glycemic control and reduced risk of hypoglycemia than NPH insulin in patients with type 1 diabetes on a basal–bolus regimen with premeal insulin aspart. Diabetes Care.

[b30] Robertson KJ, Schoenle E, Gucev Z, Mordhorst L, Gall MA, Ludvigsson J (2007). Insulin detemir compared with NPH insulin in children and adolescents with Type 1 diabetes. Diabet Med.

[b31] Kilpatrick ES, Rigby AS, Atkin SL (2008). A1c variability and the risk of microvascular complications in type 1 diabetes: data from the DCCT. Diabetes Care.

[b32] Barnard K, Thomas S, Royle P, Noyles K, Waugh N (2010). Fear of hypoglycaemia in parents of young children with type 1 diabetes: a systematic review. BMC Pediatr.

[b33] Hermansen K, Davies M, Derezinski T, Martinez RG, Clauson P, Home P (2006). A 26-week, randomized, parallel, treat-to-target trial comparing insulin detemir with NPH insulin as add-on therapy to oral glucose-lowering drugs in insulin-naive people with type 2 diabetes. Diabetes Care.

[b34] Polonsky WH, Anderson BJ, Lohrer PA, Aponte JE, Jacobson AM, Cole CF (1994). Insulin omission in women with IDDM. Diabetes Care.

[b35] Haagen BF (2011). Insulin omission. A troubling trend among adolescent girls. J Psychosoc Nurs Ment Health Serv.

