# Supplementary material for: Insulin analogues in children with Type 1 diabetes: a 52-week randomized clinical trial
Source: Diabet Med. 2013 Jan 21;30(2):216–25. doi: 10.1111/dme.12041 (PMC3579233; doi:10.1111/dme.12041)
Supplement: Table S1 — Summary of adverse events. [file dme0030-0216-sd1.doc]

**Supplemental Table** Summary of adverse events

|  | IDet | | | | NPH | | | |
| --- | --- | --- | --- | --- | --- | --- | --- | --- |
|  | Number | (%) | Events | Rate | Number | (%) | Events | Rate |
|  |  |  |  |  |  |  |  |  |
| Number of subjects | 177 |  |  |  | 170 |  |  |  |
| Total exposure (years) | 168.4 |  |  |  | 163.5 |  |  |  |
| All adverse events | 132 | 74.6 | 537 | 3190 | 135 | 79.4 | 554 | 3388 |
| Serious adverse events | 14 | 7.9 | 16 | 95 | 20 | 11.8 | 24 | 147 |
| Most frequent SAEs |  |  |  |  |  |  |  |  |
| Diabetic ketoacidosis | 3 | 1.7 | 3 | 17.8 | 4 | 2.4 | 4 | 24.5 |
| Hypoglycaemia | 1 | 0.6 | 1 | 5.9 | 3 | 1.8 | 3 | 18.3 |
| Gastroenteritis | 4 | 2.3 | 4 | 23.8 | 2 | 1.2 | 2 | 12.2 |
| Most frequent AEs |  |  |  |  |  |  |  |  |
| Nasopharyngitis | 75 | 42.4 | 147 | 873.2 | 81 | 47.6 | 179 | 1094.8 |
| Pharyngitis | 19 | 10.7 | 29 | 172.3 | 15 | 8.8 | 16 | 97.9 |
| URTI | 18 | 10.2 | 32 | 190.1 | 16 | 9.4 | 32 | 195.7 |
| Headache | 26 | 14.7 | 65 | 386.1 | 23 | 13.5 | 44 | 269.1 |
| Gastroenteritis | 18 | 10.2 | 24 | 142.6 | 14 | 8.2 | 15 | 91.7 |
| Influenza | 10 | 5.6 | 14 | 83.2 | 18 | 10.6 | 25 | 152.9 |
| Adverse events by severity |  |  |  |  |  |  |  |  |
| Mild | 124 | 70.1 | 466 | 2768 | 131 | 77.1 | 447 | 2734 |
| Moderate | 39 | 22.0 | 64 | 380 | 46 | 27.1 | 92 | 563 |
| Severe | 5 | 2.8 | 7 | 42 | 14 | 8.2 | 15 | 92 |
| Adverse events by relationship |  |  |  |  |  |  |  |  |
| Possible | 6 | 3.4 | 7 | 42 | 8 | 4.7 | 10 | 61 |
| Probable | 15 | 8.5 | 16 | 65 | 10 | 5.9 | 12 | 73 |
| Unlikely | 127 | 71.8 | 514 | 3053 | 135 | 79.4 | 531 | 3248 |
| Adverse events probably or possibly related to trial product |  |  |  |  |  |  |  |  |
| Injection-site disorders | 8 | 4.5 | 8 | 47.5 | 5 | 2.9 | 6 | 36.7 |
| Skin disorders | 7 | 4.0 | 8 | 47.5 | 4 | 2.4 | 4 | 24.5 |
| Metabolic disorders | 1 | 0.6 | 1 | 5.9 | 6 | 3.5 | 6 | 36.7 |

%, percentage of subjects; AE, adverse event; E, number of episodes; IDet, insulin detemir; N, number of subjects; NPH, neutral protamine Hagedorn; R, rate = number of episodes per 1000 subject-year of exposure; SAE, serious adverse event; URTI, upper respiratory tract infection
